# Anxiety, depression and associated factors among caretakers of children with atopic dermatitis

**DOI:** 10.1186/s12991-022-00389-z

**Published:** 2022-04-28

**Authors:** Wei Su, Hongguang Chen, Ying Gao, Qin Qin, Boqi Liu, Wei Deng, Yuhan Wang, Gaolei Zhang, Xiaoyan Liu

**Affiliations:** 1 Department of Dermatology, Capital Institute of Pediatrics, No. 2 YaBao Street, ChaoYang District, Beijing, 100020 China; 2 Key Laboratory of Mental Health, Peking University Sixth Hospital, National Clinical Research Center for Mental Disorders (Peking University Sixth Hospital), Peking University Institute of Mental Health, Ministry of Health (Peking University), Beijing, China; 3 Department of Neurology, Capital Institute of Pediatrics, Beijing, China

**Keywords:** Anxiety, Atopic dermatitis, Caretakers, Depression, Medication compliance

## Abstract

**Background:**

The prevalence of anxiety and depression symptoms among caretakers of children with atopic dermatitis (AD) and associated factors is unclear. The study was designed to explore anxiety and depression symptoms among caretakers of AD, and screen factors associated with anxiety and depression symptoms.

**Methods:**

A total of 901 children with AD and their caretakers were continuously enrolled and interviewed at dermatology department of Capital Institute of Pediatrics, Beijing, China. Children’s medication was administered by their caretakers. Caretakers’ anxiety and depression symptoms were evaluated by Hamilton Depression Rating Scale-17 and Hamilton Depression Rating Scale, while medication compliance was evaluated and divided into poor (< 6 points), moderate (6–7 points), and good (> 7 points) by Morisky Medication Adherence Scale. Multilevel ordered logistic regression was used to screen factors associated with caretakers’ anxiety and depression.

**Results:**

Among caretakers, 41.5% had anxiety symptoms, 39.6% had depression symptoms, 51.4% have any of the two and 29.7% had both of the two. Factors associated with caretakers’ anxiety symptoms included longer duration of the illness (OR, 0.99, 95% CI 0.98–0.99) and taking care of children with severe AD (OR, 2.55, 95% CI 1.43–4.55). Factors associated with caretakers’ depression symptoms included higher educational level (OR, 0.56, 95% CI 0.39–0.80), taking care of children with moderate (OR, 2.01, 95% CI 1.15–3.50) and severe AD (OR, 3.99, 95% CI 2.10–7.59) and poor medication compliance (OR, 3.45, 95% CI 1.13–10.56).

**Conclusions:**

Prevalence of anxiety and depression symptoms among caretakers of AD were high. Attention should be paid to caretakers of AD at higher risk for those psychological problems.

## Background

Pediatric atopic dermatitis (AD) is a multi-gene chronic, recurrent, inflammatory skin disease [1, 2]. Its clinical manifestations include eczema-like skin lesions, severe itching, and sleep disruption, which seriously affect the quality of life and psychological conditions of children and their caretakers [1, 3, 4]. Over the past 30 years, a 2- to 3-fold increase in pediatric AD has been reported [2, 5]. In China, the prevalence was 12.9% [6]. Previous studies had showed that AD was associated with significantly increased anxiety and depression in adults and children [7, 8]. A systematic review and meta-analysis found that prevalence of any depression was higher in persons with versus without AD (20.1% vs. 14.8%) with pooled OR 1.71 [9]. It was also reported that AD was associated with significantly higher rates of parental depression [9]. For children with AD, their medications are administered by their caretakers. Their attitudes, behaviors and psychological health would be crucial for medication compliance and therapeutic effectiveness. However, little is known about the psychological problems among caretakers of children with AD in China. This study was designed to explore the common psychological problems among caretakers of children with AD, such as anxiety and depression, and screen associated factors.

## Methods

### Participants and study design

**Fig. 1:**
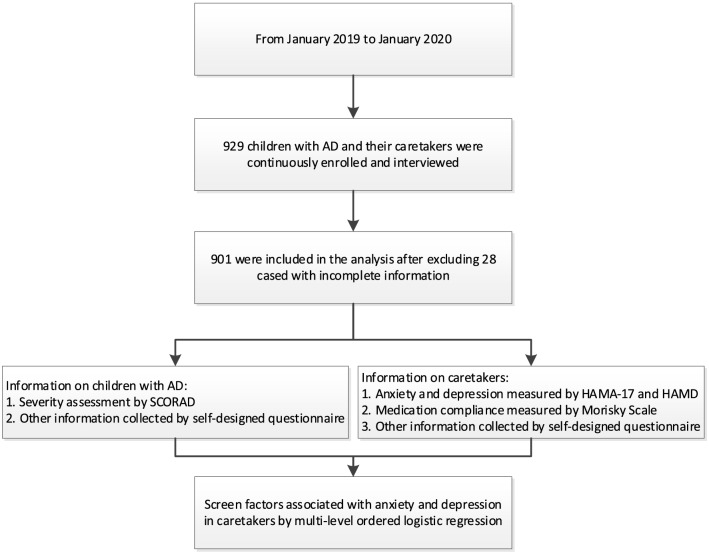
Flowchart of materials and methods

From January 2019 to January 2020, a total of 929 cases diagnosed with AD according to Williams’ [10, 11] diagnostic criteria were continuously enrolled and interviewed at the dermatology clinic of the Capital Institute of Pediatrics (see Fig. 1). Finally, 901 cases were included in the analysis after excluding 28 cases with incomplete information. The age ranges of children included were 2–18 years. Cases with other chronic diseases or congenital malformations, with a history of hospitalization within nearly half a year as well as caretakers with a history of mental illness and other chronic medical history were excluded. A self-designed questionnaire was used to collect general information on AD cases and their caretakers, including children’s age, gender and disease duration, caretakers’ education level, occupation, residence and ethnicity. This survey only investigated one of the caretakers who lived with their children and took care of their daily life.

The severity of the disease was scored according to the scoring atopic dermatitis (SCORAD) standard [12], which was divided into three grades: mild (0–24 points), moderate (25–50 points), and severe (> 50 points). The diagnosis and SCORAD score were measured by the same dermatologist in charge.

Anxiety and depression symptoms of the caretakers were measured by trained medical staff. Anxiety symptoms were assessed by the Hamilton Anxiety Scale (HAMA-17) [13, 14], which was divided into five grades: extremely severe (≥ 29 points), severe (≥ 21 and < 29 points), moderate (≥ 14 and < 21 points), mild (≥ 7 and < 14 points), and none (< 7 points). Depression symptoms were assessed by the Hamilton Depression Scale (HAMD) [15], which was divided into four grades: severe (≥ 25 points), moderate (≥ 18 and < 25 points), mild (≥ 7 and < 18 points), and none (< 7 points).

For children with AD, their medications are administered by their caretakers. The medication compliance of the caretakers was evaluated according to Morisky’s scale, which was divided into three grades: poor (< 6 points), moderate (6–7 points), and good (> 7 points). A self-report questionnaire was used to collect the reasons for poor compliance.

This article adhered to the STROBE guidelines and this study was approved by the Ethics Committee of the Capital Institute of Pediatrics. Written informed content was obtained from all the cases and their caretakers.

### Statistical analysis

The detection rates were used to describe the prevalence of anxiety and depression symptoms among caretakers. Proportions were used to describe the distributions of potential factors associated with anxiety and depression symptoms of caretakers. Nonparametric rank sum test, Spearman’s rank correlation and Kruskal–Wallis rank test were used to compare the prevalence distributions of anxiety and depression symptoms according to types of grouped variables. The data collected in this study had a two-level structure that the sources of the subjects were from Beijing and other cities. The allocation of medical resources and economic level between Beijing and other cities were completely different. Therefore, two-level mixed ordered logistic regression was used to evaluate the factors associated with anxiety and depression and calculate the 95% confidence interval. All analyses with *P* < 0.05 were considered statistically significant. All data were entered into EpiData 3.1 and analyzed using Stata software.

## Results

### Demographic characteristics

Among the children with AD, 399 (44.3%)were boys with a median age of 6.2 years (interquartile range, 5.9–6.7) and 502 (55.7%) were girls with a median age of 5.9 years (interquartile range, 5.6–6.2). Among the caretakers, the median age was 35 (interquartile range, 32–39) years, 891 (98.9%) were mothers, 167 (18.5%) were full-time caretakers, 390 (43.3%) had bachelor degree and 517 (57.4%) were from Beijing. The average duration of AD was 54 months (interquartile range, 36–84 months). In the severity classification of children with AD, 91 cases (10.1%) were rated as mild, 673 cases (74.7%) as moderate, and 137 cases (15.2%) as serious.

### Detection rates of anxiety and depression symptoms in the caretakers

The detection rates of anxiety symptoms, depression symptoms, and any of and comorbidity of the two were 41.5%, 39.6%, 51.4% and 29.7%, respectively (see Table 1). Among the caretakers with anxiety or depression symptoms, those with mild anxiety and mild depression accounted for 64% and 88%, respectively.

**Table 1:** Prevalence of anxiety and depression in caretakers of children with AD

Variables	Severity	Number	Prevalence (%)
Anxiety	Very severe	14	1.6
	Severe	33	3.7
	Moderate	86	9.5
	Mild	241	26.7
	Normal	527	58.5
Depression	Severe	5	0.6
	Moderate	38	4.2
	Mild	314	34.9
	Normal	544	60.4
Any of the two		463	51.4
Both of the two		268	29.7

### Distributions of anxiety and depression symptoms among caretakers

Higher proportion of those with anxiety symptoms were found among caretakers who took care of children with serious AD, with poor medication compliance, and from other cities other than Beijing (*P* < 0.05, see Table 2). However, there were no statistically significant differences in anxiety symptoms with regard to the age of onset, duration of illness, working status, education level and ethnicity (*P* > 0.05).

**Table 2:** Distribution of anxiety and depression symptoms in caretakers of children with AD

Variables	Anxiety symptoms					*P*	Depression symptoms				*P*
	Very severe	Severe	Moderate	Mild	Normal		Severe	Moderate	Mild	Normal	
Duration of illness											
Median (interquartile range)	50 (40–86)	48 (25–79)	52 (30–78)	51 (33–79)	59 (36–84)	0.175	60 (31–88)	50 (31–90)	52 (31–80)	56 (36–84)	0.533
Age onset											
Median (interquartile range)	5 (5–8)	5 (4–8)	6 (5–9)	6 (4–8)	6 (4–9)	0.687	7 (4–8)	5 (4–11)	6 (4–8)	6 (4–9)	0.944
Working status											
Stay-at-home	11 (1.5)	20 (2.7)	69 (9.4)	196 (26.7)	438 (59.7)	0.055	4 (0.5)	28 (3.8)	246 (33.5)	456 (62.1)	0.021
Working	3 (1.8)	13 (7.8)	17 (10.2)	45 (26.9)	89 (53.3)		1 (0.6)	10 (6.0)	68 (40.7)	88 (52.7)	
Education level											
High school or below	3 (1.5)	11 (5.5)	23 (11.6)	56 (28.1)	106 (53.3)	0.057	2 (1.0)	15 (7.5)	85 (42.7)	97 (48.7)	< 0.001
College degree or above	11 (1.6)	22 (3.1)	63 (9.0)	185 (26.4)	421 (60.0)		3 (0.4)	23 (3.3)	229 (32.6)	447 (63.7)	
Ethnicity											
Han	14 (1.6)	30 (3.5)	81 (9.3)	232 (26.7)	511 (58.9)	0.155	5 (0.6)	35 (4)	304 (35)	524 (60.4)	0.865
Others	0 (0.0)	3 (9.1)	5 (15.2)	9 (27.3)	16 (48.5)		0 (0.0)	3 (9.1)	10 (30.3)	20 (60.6)	
Severity of the AD											
Mild	3 (3.3)	3 (3.3)	5 (5.5)	19 (20.9)	61 (67.0)	0.003	0 (0.0)	3 (3.3)	19 (20.9)	69 (75.8)	< 0.001
Moderate	9 (1.3)	22 (3.3)	64 (9.5)	177 (26.3)	401 (59.6)		3 (0.4)	27 (4.0)	231 (34.3)	412 (61.2)	
Severe	2 (1.5)	8 (5.8)	17 (12.4)	45 (32.8)	65 (47.4)		2 (1.5)	8 (5.8)	64 (46.7)	63 (46.0)	
Medication compliance											
Good	0 (0.0)	0 (0.0)	2 (8.3)	6 (25.0)	16 (66.7)	< 0.001	0 (0.0)	1 (4.2)	4 (16.7)	19 (79.2)	< 0.001
Moderate	2 (1.1)	7 (3.7)	11 (5.8)	35 (18.4)	135 (71.1)		0 (0.0)	4 (2.1)	49 (25.8)	137 (72.1)	
Poor	12 (1.7)	26 (3.8)	73 (10.6)	200 (29.2)	375 (54.7)		5 (0.7)	33 (4.8)	261 (38.0)	387 (56.4)	
Residence											
Beijing	7 (1.4)	14 (2.7)	41 (7.9)	127 (24.6)	328 (63.4)	< 0.001	1 (0.2)	13(2.5)	160 (30.9)	343 (66.3)	< 0.001
Non-Beijing	7 (1.8)	19 (4.9)	45 (11.7)	114 (29.7)	199 (51.8)		4 (1.0)	25 (6.5)	154 (40.1)	201 (52.3)	

Higher proportion of those with depression symptoms were found among caretakers who had to work in addition to take care of their children, with low educational background, taking care of children with serious AD, with poor medication compliance, and from other cities other than Beijing. There were no statistically significant differences in depression symptoms with regard to the age of onset and the duration of illness (*P* > 0.05). The main reason for poor compliance was fear of side-effects (45.4%), followed by worries about dependence (16.5%), especially corticosteroids medications.

### Factors associated with anxiety and depression symptoms among caretakers

For the analysis on anxiety symptoms, the estimate of variance in level 2 (City) was 0.01 with the standard error of 0.02. A likelihood ratio test comparing the model with the one-level ordered logistic regression did not favor the random-intercept model with *P* = 0.187. For the analysis on depression symptoms, the estimate of variance in level 2 (City) was 0.03 with the standard error of 0.04. A likelihood ratio test comparing the model with the one-level ordered logistic regression favored the random-intercept model with *P* = 0.049. Therefore, two-level mixed ordered logistic regression was used to screen factors associated with depression symptoms and one-level ordered logistic regression was used for analysis on factors associated with anxiety symptoms.

Factors associated with caretakers’ anxiety symptoms included longer duration of the illness (OR, 0.99, 95% CI 0.98–0.99) and taking care of children with severe AD (OR, 2.55, 95% CI 1.43–4.55) (see Table 3). Factors associated with caretakers’ depression symptoms included higher educational level (OR, 0.56, 95% CI 0.39–0.80), taking care of children with moderate (OR, 2.01, 95% CI 1.15–3.50) and severe AD (OR, 3.99, 95% CI 2.10–7.59) and poor medication compliance (OR, 3.45, 95% CI 1.13–10.56).

**Table 3:** Factors associated with anxiety and depression symptoms in caretakers of children with AD^†^

Variables	Anxiety symptoms		Depression symptoms	
	Adjusted OR (95% CI)	*P*	Adjusted OR (95% CI)	*P*
Duration of illness				
Median (interquartile range)	0.99 (0.98–0.99)	0.005	0.99 (0.99–1.00)	0.052
Age onset				
Median (interquartile range)	1.08 (0.99–1.16)	0.051	1.06 (0.98–1.15)	0.126
Working status				
Stay-at-home	1		1	
Working	1.30 (0.91–1.85)	0.143	1.22 (0.84–1.76)	0.295
Education level				
High school or below	1		1	
College degree or above	0.83 (0.59–1.17)	0.297	0.56 (0.39–0.80)	0.001
Ethnicity				
Han	1		1	
Others	1.85 (0.84–4.07)	0.126	0.99 (0.42–2.36)	0.987
Severity of the AD				
Mild	1		1	
Moderate	1.38 (0.84–2.28)	0.201	2.01 (1.15–3.50)	0.014
Severe	2.55 (1.43–4.55)	0.001	3.99 (2.10–7.59)	< 0.001
Medication compliance				
Good	1		1	
Moderate	0.87 (0.34–2.27)	0.783	1.56 (0.49–4.96)	0.454
Poor	1.90 (0.77–4.68)	0.166	3.45 (1.13–10.56)	0.030
Level two (residence)				
Variance	N/A		0.032	

The estimate of variance in level 2 was 0.01 with the standard error of 0.02 in the two-level (Beijing vs. non-Beijing) ordered logistic regression analysis for anxiety. An LR test comparing the model with the one-level survival model didn’t favor the random-intercept model with *P* > 0.05. The estimate of variance in level 2 was 0.32 with the standard error of 0.04 in the two-level ordered logistic regression analysis for depression. An LR test comparing the model with the one-level survival model favored the random-intercept model with *P* < 0.05

## Discussion

Our study initially explored the psychological problems of caretakers of children with AD and their associated factors in China. The study found that among the investigated caretakers, 41.5% had anxiety symptoms, 39.6% had depression symptoms, 29.7% had both of the two and 51.4% have any of the two, and most experienced mild symptoms of anxiety and depression. The detection rates of depression and anxiety symptom in caretakers were higher than the findings of other studies on parental depression and anxiety [9, 16]. Both the detection rates of anxiety and depression symptoms in caretakers of children with AD were higher than those reported from children and adult AD themselves [7, 17–20], which may be related to the specific disease characteristics of AD and more intimate parent–child relationship in China [21]. It was reported that compared with looking after a child with chronic asthma, caring for a child with chronic atopic eczema was associated with greater parental sleep disturbances [22]. Because of improper or irregular medication, AD had frequent relapses and a protracted course. All these factors might increase the risk of psychological problems in caretakers [23].

This study also found that factors associated with anxiety and depression symptoms were not identical. Only two factors were found to be associated with higher risk of anxiety symptoms. Longer duration of the disease turned out to be a protective factor for anxiety depressions. Otherwise, caretakers were more likely to have anxiety at the onset of AD. The reason might be related to the lack of understanding of this sudden onset disease, especially when there was no short-term cure. It was common sense that caretakers suffered from higher risk of anxiety symptoms when they took care of children with serious AD. As for that of depression symptoms, higher education level could reduce the risk for depression symptoms, which was consistent with previous finding. It was reported that higher educational level seemed to have a protective effect against anxiety and depression, which accumulated throughout life [24]. Similar to that of anxiety, caretakers were more likely to have depression when their children were moderate and serious AD. Psychosocial stressors might contribute to this. Previous study reported that more severe childhood AD led to increased rates of parental stress, parental depression, worsening behavioral problems in children, and difficulty managing AD [25, 26]. Poor medication compliance is believed to be one of the main factors associated with poor treatment outcomes, recurrent attacks and the occurrence of symptoms in children with AD. Unlike anxiety, poor medication compliance was found to be associated with depression symptoms. In this study, poor compliance was particularly highlighted in their fear of side-effects of topical corticosteroid. It was reported that 67.5% of the caretakers showed topical corticosteroid phobia [27]. A study of 579 parents of children with AD had also reported that significant problems associated with topical corticosteroid use and major difficulties managing AD in their children [28]. Other inappropriate uses of medication included not using medication according to the course of treatment, and using only non-corticosteroids topical medication. Corticosteroids are the first-line treatment regime and play an important role in treatment of children with AD. Caretakers often misinterpret the pharmacological characteristics and application value of corticosteroids as well as their fear of side-effects and dependence. In clinical practice, application value of the pharmacological properties of medicines such as corticosteroids should be explained as detailed as possible to the children and their caretakers so as to improve the medication compliance and eliminate caretakers’ depression symptoms.

However, this study has some limitations. It was a cross-sectional study, which did not enable the identification of causal relationships. This study only investigated the symptoms of anxiety and depression, and not diagnosis of psychological disorders.

## Conclusions

The prevalence of anxiety and depression symptoms among caretakers of children with AD was high. We suggest that health education should be emphasized to clarify the nature of AD and the value of medication compliance for treatment of AD. Moreover, it is necessary to pay attention to the psychological problems of caretakers of children with AD in clinical practices.

## Data Availability

The datasets used and/or analyzed during the current study are available from the corresponding author on reasonable request.

## Abbreviations

AD: Atopic dermatitis
HAMA: Hamilton Anxiety Scale
HAMD: Hamilton Depression Scale
OR: Odds ratio
95%CI: 95% confidence interval
SCORAD: Scoring atopic dermatitis
